# Mortality Statistics

**Published:** 1874-07

**Authors:** 


### ***Chicago Mortality Report for May, 1874.***

MORTALITY IN MONTH OF MAY, 1874.

| | | | |
|---|---|---|---|
| Accident, by burns | 1 | Abscess, hepatic | 2 |
| " by being crushed | 1 | Ascites | 1 |
| " by drowning | 4 | Angina pectoris | 1 |
| " by blast | 1 | Aphthæ | 1 |
| " by fall | 1 | Apoplexy | 7 |
| " kicked by mule | 1 | Asphyxia | 2 |
| " thrown from buggy | 1 | Asthma | 1 |
| " run over by wagon | 1 | Atelectasis pulmonum | 1 |
| " overdose morphine | 1 | Bowels, obstruction of | 2 |
| " by railroad | 2 | Brain, compression of | 1 |
| " by shooting | 1 | " and lungs, tuberculosis of | 1 |
| " by circular saw | 1 | " congestion of | 7 |
| " by scalding | 1 | " disease of | 1 |

| | |
|---|---|
| Brain, hemorrhage of | 1 |
| " inflammation of | 11 |
| " softening of | 4 |
| Bronchitis | 6 |
| Cancer of breast | 1 |
| " bladder | 1 |
| " maxilla | 1 |
| " mesentery | 1 |
| " liver | 1 |
| " pelvis | 1 |
| " stomach | 5 |
| " uterus | 1 |
| Cholera Infantum | 4 |
| " Morbus | 1 |
| Consumption | 54 |
| Convulsions | 57 |
| Croup | 4 |
| " membranous | 3 |
| Cyanosis | 1 |
| Cynanche trachealis | 1 |
| Debility, general | 3 |
| Delirium tremens | 1 |
| Deficient development | 1 |
| Diabetes | 1 |
| Diarrhœa | 2 |
| Diphtheria | 4 |
| Dropsy, general | 4 |
| " of chest | 1 |
| " cardiac | 1 |
| " of abdomen | 1 |
| Dysentery | 4 |
| Embolism | 1 |
| Endo pericarditis | 1 |
| " carditis | 1 |
| Enteritis | 21 |
| Epilepsy | 3 |
| Exhaustion | 4 |
| Erysipelas | 7 |
| Fever, puerperal | 4 |
| " remittent | 1 |
| " scarlet | 6 |
| " typhoid | 5 |
| Gastritis | 6 |
| Gastro enteritis | 3 |
| Hæmoptysis | 1 |
| Hemorrhage, cerebral | 1 |
| Heart disease | 3 |
| " dropsy of | 1 |
| " fatty degeneration of | 1 |
| " paralysis of | 1 |
| " organic disease of | 3 |
| " rheumatism of | 1 |
| " valvular disease of | 3 |
| Hernia, incarcerated | 1 |
| Hepatitis | 2 |
| Hydrophobia | 1 |
| Hydrocephalus | 12 |
| Hernia, strangulated | 1 |
| Hydro pericarditis | 1 |
| Inanition | 8 |
| Intemperance | 2 |
| Kidneys, Bright's disease of | 8 |
| Laryngitis | 1 |
| Liver, cirrhosis of | 1 |
| " induration of | 1 |
| " abscess of | 1 |
| Lungs, congestion of | 13 |
| " emphysema | 1 |
| " paralysis of | 1 |
| " hemorrhage of | 1 |
| " œdema of | 1 |
| Malformation | 1 |
| Metro peritonitis | 1 |
| Myelitis | 1 |
| Measles | 2 |
| Meningitis | 13 |
| " cerebro spinal | 7 |
| " tubercular | 1 |
| Metritis, puerperal | 1 |
| Metro phlebitis | 1 |
| Œdema pulmonum | 1 |
| Old Age | 9 |
| Paralysis | 3 |
| Pelvic cellulitis | 1 |
| Pericarditis | 4 |
| Peritonitis | 4 |
| " puerperal | 4 |
| Pneumonia | 23 |
| " typhoid | 3 |
| Pleurisy | 5 |
| Prurigo | 1 |
| Pyæmia | 3 |
| Scrofula | 2 |
| Septicæmia | 1 |
| Small-Pox | 14 |
| Suicide, drowning | 1 |
| " hanging | 1 |
| " morphine | 3 |
| " shooting | 2 |
| Spine, disease of | 1 |
| Stomach, hemorrhage of | 1 |
| Teething | 3 |
| Tetanus | 1 |
| Tumor of abdomen | 1 |
| Whooping Cough | 11 |
| Total | 507 |

Premature births, 15 ; Still births, 57. Total 72

COMPARISON.

| | |
|---|---|
| Deaths in month of May, 1874 | 507 |
| " " April, 1874 | 486 |
| Increase | 21 |
| Deaths in month of May, 1873 | 630 |
| Decrease | 123 |

AGES.

| | | | |
|---|---|---|---|
| Under one year | 167 | Forty years to fifty | 36 |
| One year to two | 33 | Fifty " " sixty | 36 |
| Two years to three | 14 | Sixty " " seventy | 24 |
| Three " " four | 5 | Seventy " " eighty | 18 |
| Four " " five | 5 | Eighty " " ninety | 5 |
| Five " " ten | 23 | Ninety " " one hundred | -- |
| Ten " " twenty | 25 | | |
| Twenty " " thirty | 49 | Total | 507 |
| Thirty " " forty | 67 | | |
| Colored | 7 | Males | 283 |
| White | 500 | Females | 224 |
| Total | 507 | Total | 507 |

Married, 198; Single, 309. Total 507

NATIVITIES.

| | | | |
|---|---|---|---|
| Austria | 1 | Germany | 69 |
| Bohemia | 8 | Holland | 1 |
| Canada | 14 | Ireland | 44 |
| China | 1 | Norway | 11 |
| Native—Chicago | 49 | Poland | 2 |
| Foreign, " | 164 | Scotland | 5 |
| United States, other parts | 101 | Sweden | 5 |
| Denmark | 1 | Switzerland | 2 |
| England | 18 | Wales | 1 |
| France | 3 | Unknown | 7 |

Deaths daily, 16½. Mean thermometer, 58.3°. Rain fall, 2.09 inches.

MORTALITY BY WARDS, ETC.

| Wards. | No. Deaths. | Pop. in 1872. | Percentage. |
|---|---|---|---|
| 1 | 1 | 398 | one death in ---- |
| 2 | 2 | 2,174 | " " " ---- |
| 3 | 15 | 19,157 | " " " 1,277 |
| 4 | 7 | 16,832 | " " " 2,405 |
| 5 | 19 | 18,564 | " " " 977 |
| 6 | 38 | 31,371 | " " " 823 |
| 7 | 32 | 27,644 | " " " 864 |
| 8 | 32 | 30,253 | " " " 945 |
| 9 | 36 | 30,032 | " " " 834 |
| 10 | 13 | 16,624 | " " " 1,278 |
| 11 | 11 | 18,340 | " " " 1,667 |
| 12 | 17 | 20,876 | " " " 1,228 |
| 13 | 9 | 14,636 | " " " 1,626 |
| 14 | 19 | 15,892 | " " " 836 |
| 15 | 64 | 40,047 | " " " 626 |
| 16 | 43 | 19,099 | " " " 444 |
| 17 | 28 | 17,513 | " " " 625 |
| 18 | 33 | 19,977 | " " " 605 |
| 19 | 4 | 2,944 | " " " ---- |
| 20 | 11 | 5,023 | " " " ---- |
| | 434 | Ratio of deaths to population in 1872, one death in 721. | |

| | | | |
|---|---|---|---|
| No. deaths in Wards | 434 | Immigrants | 1 |
| Accidents | 17 | Mercy Hospital | 1 |
| Alexian Brothers Hospital | 1 | Protestant Orphan Asylum | 1 |
| Bridewell | 2 | Small-Pox Hospital | 2 |
| County Hospital | 12 | St. Joseph's Hospital | 5 |
| Foundlings' Home | 19 | St. Luke's Hospital | 2 |
| Hahneman Hospital | 1 | Suicides | 6 |
| Hospital for Women and Children | 3 | Total | 507 |

CASES OF SMALL-POX REPORTED DURING MAY, 1874.

| Wards. | No. Cases. | Wards. | No. Cases. | Wards. | No. Cases. |
|---|---|---|---|---|---|
| 1 | -- | 8 | 1 | 15 | 6 |
| 2 | -- | 9 | 3 | 16 | 6 |
| 3 | -- | 10 | 1 | 17 | 4 |
| 4 | 2 | 11 | -- | 18 | 12 |
| 5 | 1 | 12 | -- | 19 | 2 |
| 6 | 6 | 13 | -- | 20 | 4 |
| 7 | 5 | 14 | -- | Hospitals, | 3 |
| Total | | | | | 56 |

| | |
|---|---|
| Cases reported during May, 1874 | 56 |
| " " " April, 1874 | 63 |
| Decrease | 7 |
| Cases reported during May, 1873 | |
| Decrease | 14 |

